# Novel Computerized Method for Automated Determination of Ventilatory Threshold and Respiratory Compensation Point

**DOI:** 10.3389/fphys.2021.782167

**Published:** 2021-12-17

**Authors:** Kyoung Jae Kim, Eric Rivas, Brian Prejean, Dillon Frisco, Millennia Young, Meghan Downs

**Affiliations:** ^1^KBR, Houston, TX, United States; ^2^JES Technologies, Houston, TX, United States; ^3^NASA Johnson Space Center, Houston, TX, United States

**Keywords:** incremental exercise, noninvasive measurement, ventilatory threshold, respiratory compensation point, automated determination

## Abstract

**Introduction:** The ventilatory threshold (named as VT_1_) and the respiratory compensation point (named as VT_2_) describe prominent changes of metabolic demand and exercise intensity domains during an incremental exercise test.

**Methods:** A novel computerized method based on the optimization method was developed for automatically determining VT_1_ and VT_2_ from expired air during a progressive maximal exercise test. A total of 109 peak cycle tests were performed by members of the US astronaut corps (74 males and 35 females). We compared the automatically determined VT_1_ and VT_2_ values against the visual subjective and independent analyses of three trained evaluators. We also characterized VT_1_ and VT_2_ and the respective absolute and relative work rates and distinguished differences between sexes.

**Results:** The automated compared to the visual subjective values were analyzed for differences with *t* test, for agreement with Bland–Altman plots, and for equivalence with a two one-sided test approach. The results showed that the automated and visual subjective methods were statistically equivalent, and the proposed approach reliably determined VT_1_ and VT_2_ values. Females had lower absolute O_2_ uptake, work rate, and ventilation, and relative O_2_ uptake at VT_1_ and VT_2_ compared to men (*p* ≤ 0.04). VT_1_ and VT_2_ occurred at a greater relative percentage of their peak VO_2_ for females (67 and 88%) compared to males (55 and 74%; main effect for sex: *p* < 0.001). Overall, VT_1_ occurred at 58% of peak VO_2,_ and VT_2_ occurred at 79% of peak VO_2_ (*p* < 0.0001).

**Conclusion:** Improvements in determining of VT_1_ and VT_2_ by automated analysis are time efficient, valid, and comparable to subjective visual analysis and may provide valuable information in research and clinical practice as well as identifying exercise intensity domains of crewmembers in space.

## Introduction

Examining the ventilatory profile during incremental exercise to a maximum effort has been used to assess aerobic fitness and monitor and prescribe exercise training in athletes and clinical populations with decades of controversy (Yeh et al., 1983; Poole et al., 2021). Noninvasive measurements of the ventilatory profile are represented commonly as two inflection points: the ventilatory threshold (named as VT_1_ in this paper) and the respiratory compensation point (named as VT_2_ in this paper; Whipp et al., 1989; Cannon et al., 2009; Pallarés et al., 2016; Carriere et al., 2019; Galán-Rioja et al., 2020). Determining VT_1_ and VT_2_ during an incremental exercise test should be accompanied by a set of universally agreed upon and explicitly defined quality-control criteria (Meyer et al., 1996; Gaskill et al., 2001; Poole et al., 2016; Galán-Rioja et al., 2020). Several VT methods for detecting VT have traditionally been based on a subjective visual analysis, but this is time-consuming, requires 2–3 trained personnel with an independent reviewer and a strict set of criteria to maintain tight quality control (Caiozzo et al., 1982; Yeh et al., 1983; Gladden et al., 1985; Dickstein et al., 1990a; Shimizu et al., 1991).

VT_1_ is commonly described as the point at which pulmonary ventilation and carbon dioxide (CO_2_) output begin to increase exponentially (Whipp et al., 1989; Dennis et al., 1992). The ventilatory equivalent method has been used to identify VT_1_, which is best described as the intensity of activity that causes the first rise in the ventilatory equivalent of oxygen (O_2_) without a concurrent rise in the ventilatory equivalent of CO_2_ (Reinhard et al., 1979; Powers et al., 1984). Determining VT_1_ using the excess CO_2_ (ExCO_2_) method requires the intensity of exercise that causes an increase from steady state to an excess production of CO_2_ (Volkov et al., 1975). Likewise, the V-slope method, also commonly used method, uses points that show an increase in the slope from less than 1 to greater than 1 in the CO_2_ production (VCO_2_) by O_2_ consumption (VO_2_) data (Dickstein et al., 1990a; Ekkekakis et al., 2008) and in the minute ventilation (VE) by VCO_2_ data (Beaver et al., 1986) for locating VT_1_ and VT_2_, respectively. All computerized methods mentioned require finding the point where the slope changes markedly over the entire scattergram. However, two regression lines skim the slope of data points from left to right and stop as soon as its criterion is satisfied; thus, the algorithm for searching VT_1_ might estimate lower VO_2_ compared with the other methods depending on the amount of noise in the data (Ekkekakis et al., 2008). For the severe exercise intensity domain, VT_2_ is considered the second break point at which the partial pressure of arterial CO_2_ starts to decline during heavy exercise (Beaver et al., 1986; Nattie and Li, 2012). The 2-line regression model for detecting the slope change in the scattergram also has been used to find VT_2_ associated with VE and VCO_2_ (Volkov et al., 1975; Reinhard et al., 1979; Davis et al., 1980; Powers et al., 1984; Brooks, 1985; Beaver et al., 1986). However, a disadvantage, inherent to methods that use regression analysis during maximal exercise, is the possibility that the hyperventilation phase (VT_2_ starting point) may be partially included in the calculation (Dickstein et al., 1990b).

Exercise countermeasures are the only known way for maintaining muscle mass, strength, and cardiorespiratory fitness in crewmembers during spaceflight. It is important for exercise prescriptions to be optimized to maintain astronauts’ fitness to avoid premature physical and cognitive fatigue during performance of high-risk, mission-critical tasks. Additionally, more women are now flying into space, yet few studies about sex-specific physiologic differences in the astronaut population have been explored during and after spaceflight. A reliable and automated detection of VT_1_ and VT_2_ for prescribing exercise domains can help to quickly identify and guide individualized exercise prescriptions for the purpose of maintaining and enhancing crewmembers’ performance of submaximal extravehicular activities (EVAs) over long durations. We believe that determining both VT_1_ and VT_2_ using the proposed novel automated method and their associated absolute and relative work rates can provide valuable information regarding crewmembers’ ability to exercise (e.g., buffering capacity, lactate kinetics) on the International Space Station (ISS) and to perform EVAs and lunar exploration. Understanding where VT_1_ and VT_2_ occur can assist in prescribing exercise intensity above or below those points. Therefore, in this study, we determined the accuracy of our novel automated analysis by comparing the computerized results with subjective visual identification. We also aimed to characterize absolute and relative work rates at VT_1_ and VT_2_ and to identify if there are any differences in those values between female and male crewmembers.

## Materials and Methods

### Subjects

This study protocol was reviewed and approved by the NASA Johnson Space Center (JSC)‘s Institutional Review Board and in agreement with the Declaration of Helsinki. All participants in the study provided signatures confirming their informed consent. Data were collected from 2013 to 2019. Astronaut participants in these data were active astronauts in flight training at JSC. Exact training status, e.g., amount of time spent in physically training, is not known. A total of 109 peak cycle tests were obtained from a large astronaut database (74 males and 35 females) and used to examine the ventilatory thresholds at VT_1_ and VT_2_. The data set was separated into groups of female and males for the sex difference study (Pescatello et al., 2014). See Table 1 for mean participant characteristics and statistical significances for the overall and sex differences.

**Table 1:** Mean participant characteristics and statistical significances between groups.

Study		Age (year)	Mass (kg)	Height (cm)
Overall		41.1 ± 7.3	76.8 ± 12.4	174.8 ± 9.4
Male vs. Female	Male	41.5 ± 6.8	82.6 ± 9.9	179.1 ± 5.8
	Female	40.3 ± 8.4	64.7 ± 7.2	165.7 ± 9.0
	value of *p*	0.46	*<0.0001*	*<0.0001*

### Test Procedure and Data Collection

Cardiorespiratory fitness (peak VO_2_) was determined by a progressive, incremental graded cycle ergometer stress test to volitional exhaustion. Our graded exercise protocol was developed to measure peak VO_2_ and VT at JSC. The protocol has two versions; a nominal protocol and a light protocol conducted on the LODE Excalibur sport cycle ergometer (Lode BV, Groningen, Netherlands). The nominal protocol consisted of a cycling warm-up at 50 W for 3 min followed by stepwise increases of 25 W every minute until test termination. The light protocol consists of the same timed wattage increases (i.e., 3 min warm-up then 1-min increases), but the wattage starts at 45 W and with 15 W increases. Participants were assigned the nominal or light protocol based on body weight. Participants were assigned the light protocol if they weighed less than 65 kilograms. Eight participants fell in this category. We observed that aerobic response was sufficiently fast to adjust to the 15 or 25 W workload increases within the 1-min stage time.

Participants were instructed to maintain a cadence of 75 revolutions per minute (RPM) throughout testing. Respiratory gases were sampled per 10-s interval and analyzed using the ParvoMedics TrueOne^®^ 2400 metabolic cart. After a 30-min warm-up, O_2_ and CO_2_ gas were calibrated using known gases (16% O_2_, 4% CO_2_) and air flow was calibrated with a 3-L syringe. To ensure accuracy of indirect calorimetry, gas and flow calibration was conducted prior to every exercise test and consisted of ambient and standard gas calibration along with flow meter calibration. Calibration was accepted if new calibration parameters were within +/− 3% of previous values. Additionally, maintenance procedures were followed in accordance with manufacturer guidelines. The rating of perceived exertion (RPE; Borg scale 6–20) was measured every 2–3 min during the exercise test. Heart rate (HR) was determined from 12-lead electrocardiogram recordings throughout the test (CardioSoft CASE^®^, GE Healthcare, WI, United States). The test was considered to be maximal when at least 4 of 5 following criteria were met: (1) a respiratory exchange ratio (RER) of ≥1.10, (2) a plateau in VO_2_ with increasing workloads, (3) workload volitional fatigue (a fall of 10 RPM), (4) exercise peak HR that was within 10 beats of the age-predicted maximal HR [207–(0.67 × age)] (Gellish et al., 2007), and (5) RPE at or greater than 19. All participants reached at least 4 of the listed criteria.

In summary, exercise variables in this study were work rates (W), %Wmax (%), HR (beat/min), RER (VO_2_:VCO_2_), and VE (L/min) at the ventilatory thresholds (VT_1_ and VT_2_) and peak VO_2_ in absolute (L/min) and relative to body weight (ml/kg/min) expressions. The first 3 min of warm-up were excluded in data analysis.

### Proposed Computerized Determination of VT Values

**Figure 1:**
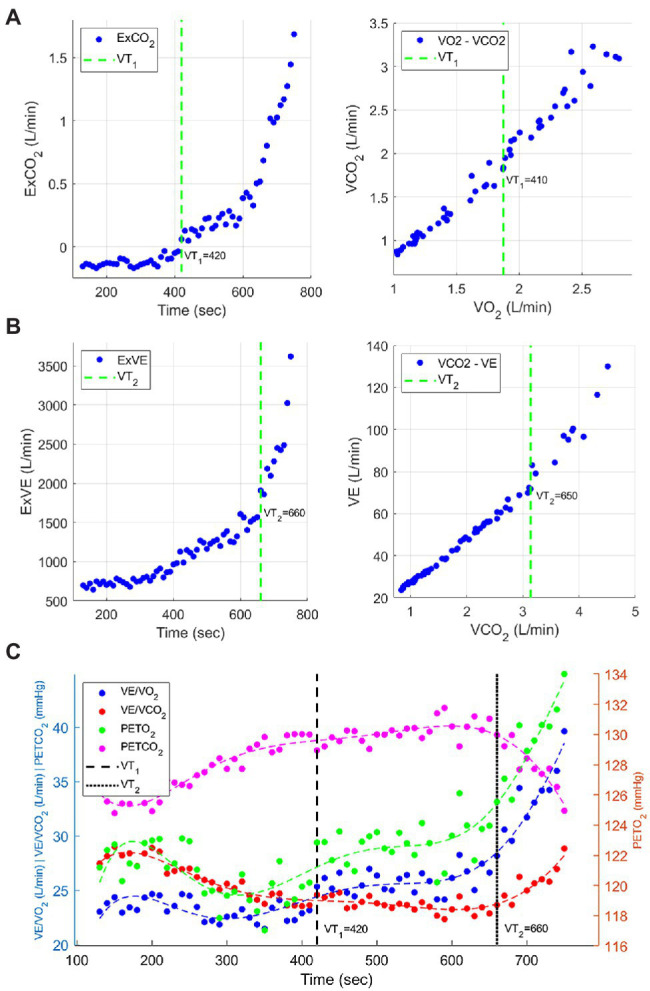
Example of determination of VT_1_ and VT_2_. **(A)** left: ExCO_2_, right: V-slope for VT_1_. **(B)** left: ExVE, right: V-slope for VT_2_. **(C)** The combined VT_1_ and VT_2_ corresponded to gas exchange data: VE/VO_2_, ventilatory equivalent for O_2_ (blue dots); VE/VCO_2_, ventilatory equivalent for CO_2_ (red dots); PETO_2_, end-tidal pressure of O_2_ (green dots); end-tidal pressure of CO_2_, PETCO_2_ (pink dots).

Detection of abrupt change in data distribution has been considered as one of the important practical problems arising in various applications (Riedel, 1994). In this study, we used a parametric global optimization method (Lavielle, 2005), which was implemented as a MATLAB function, named *findchangepts* (MATLAB^®^ R2020a, The MathWorks, Inc., Natick, MA, United States). Though the parametric global optimization method identifies a data point change most significantly over the entire scattergram, it could be common during the determination of thresholds to find that some data are indeterminate and inter-method differences are unavoidable in nature (Gaskill et al., 2001). To provide a valid and reliable process, Gaskill et al. (2001) recommended to average the combined multiple methods used to identify ventilatory threshold (Gaskill et al., 2001). Thus, we identified VT_1_ using the mean of ExCO_2_ and V-slope (Figure 1A) and detected VT_2_ using the mean of the excess minute ventilation (ExVE) method (Kim et al., 2020) and V-slope (Figure 1B). Figure 1C shows the combined VT_1_ and VT_2_ corresponded to gas exchange data: VE/VO_2_, ventilatory equivalent for O_2_ (blue dots); VE/VCO_2_, ventilatory equivalent for CO_2_ (red dots); PETO_2_, end-tidal pressure of O_2_ (green dots); end-tidal pressure of CO_2_, PETCO_2_ (pink dots). Finally, we identified the work rate associated with VT_1_ and VT_2_ and compared absolute and relative sex difference.

The ExVE is the method we proposed to identify the intensity of exercise that caused an increase from steady state to excess VE (Kim et al., 2020). Anaerobic exercise triggers a cascade of metabolic reactions in the human body (Beaver et al., 1986). At high intensity of exercise, the high-energy demand triggers a breakdown of glucose and a reduction of pyruvate. These metabolic processes produce lactate faster than the body can metabolize it. Bicarbonate buffers hydrogen H^+^ as a countermeasure when lactate flux increases faster than removal. As exercise intensity increases above this point, VCO_2_ increases and systemic pH in the bloodstream decreases as the H^+^ from the bicarbonate is not sufficiently buffered (Myers and Ashley, 1997). This decrease in blood pH triggers carotid bodies, chemoreceptor cells located in the carotid artery, to further increase VE. Therefore, VT_2_ can be determined as the point in time where VE increases to compensate for VCO_2_ being greater than VO_2_, while end-tidal CO_2_ levels are reduced (Pallarés et al., 2016). We reformulated the typical ExCO_2_ concept [e.g.,(VCO_2_^2^/VO_2_)−VCO_2_)] (Volkov et al., 1975; Gaskill et al., 2001) to the ExVE form such as (VE^2^/VCO_2_)−VE.

**Figure 2:**
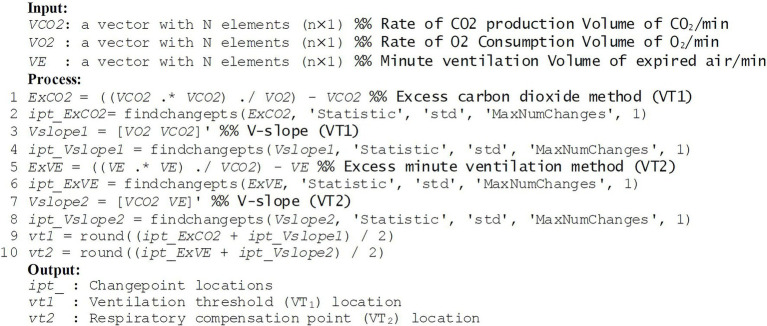
Code for the suggested approach.

Figure 2 shows the code of the procedure how we generated the input data, how we applied the *findchangepks* function to detect each changepoint location, and how we finally determined VT_1_ and VT_2_. For example, we specified “Statistic” as “std” because the “std” option detects a significant change that occurs while the standard deviation of input data distribution increases after ventilatory thresholds. Also, we specified “MaxNumChanges” as “1” to return the index of the most significant change point in the scattergrams (see green dashed lines in Figures 1A,B). The algorithm for searching might be more robust and less influenced by unexpected peak noise at the initial or end time range. It has been reported that VT_1_ and VT_2_ commonly lie at exercise intensities between 50 and 65% of VO_2_ and between 75 and 87% of VO_2_, respectively (Beaver et al., 1986; Pallarés et al., 2016; Cerezuela-Espejo et al., 2018; Galán-Rioja et al., 2020). Thus, it is recommended to use a specific range of the input signals only, for example, trimming half of the signal length (i.e., from 30th percentile to 80th percentile for VT_1_, from 50th percentile to 100th percentile for VT_2_).

### Visually Evaluated VT Values

**Figure 3:**
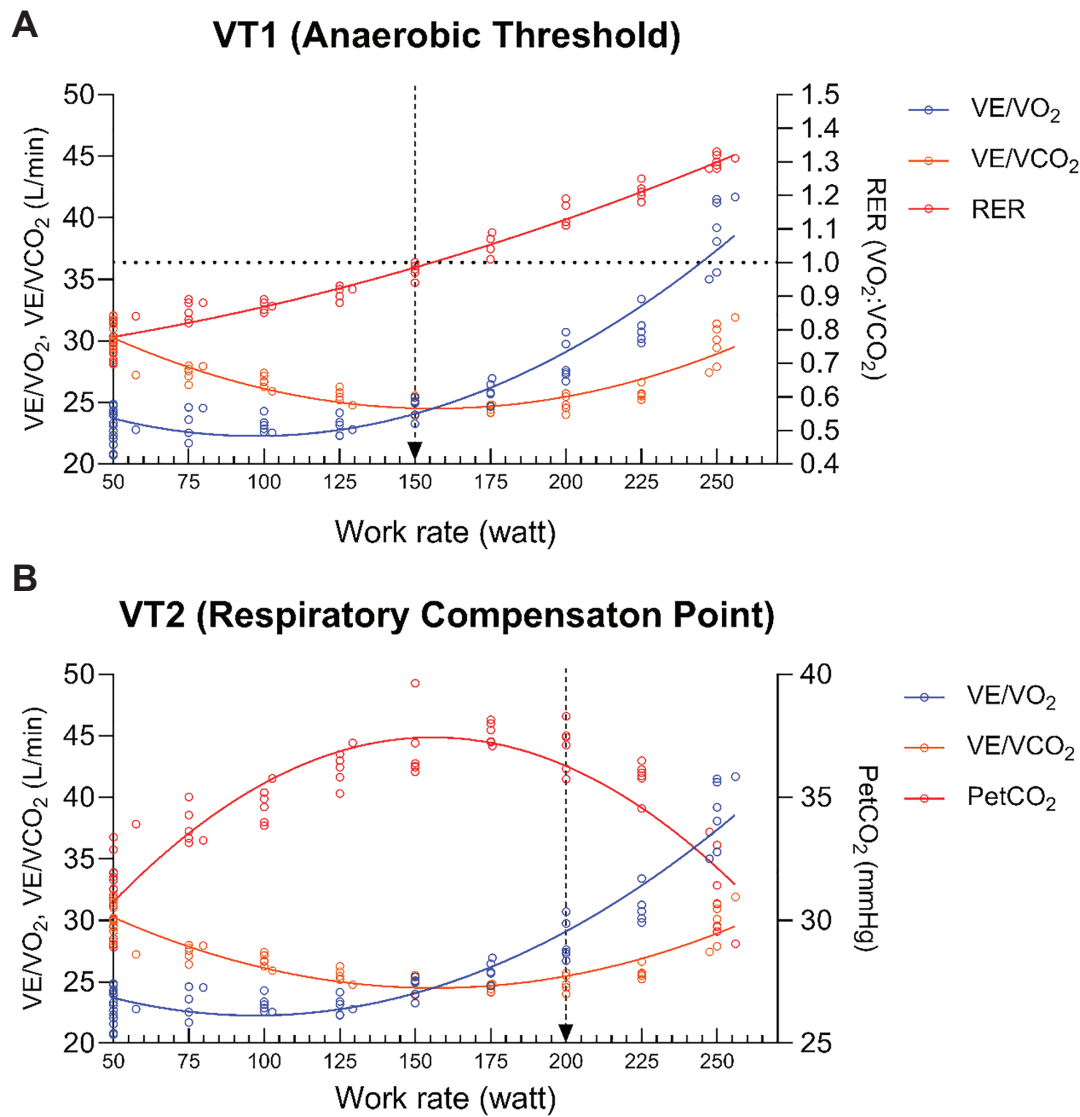
Visual determination of VT_1_ **(A)** and VT_2_ **(B)**. The arrows designate the choice of VT_1_ and VT_2_ in these sample graphs from one subject.

For the validation study, three trained evaluators independently and randomly evaluated the graphs of the data to determine VT_1_ and VT_2_ values. For each determination, graphs were visually evaluated for the assessment of change in data distribution. Specifically, for VT_1_, evaluators assessed the intensity of activity that causes the first sustained rise in the VE/VO_2_ without a concurrent rise in the ventilatory VE/VCO_2_ (Figure 3A). The rise in VE/VO_2_ is in concurrence with RER reaching 1.0. For VT_2_, evaluators assessed increase in both the VE/VO_2_ and VE/VCO_2_ (Figure 3B). This rise in VE/VO_2_ and VE/VCO_2_ is in concurrence with a decrease in PetCO_2_ (5). A detailed protocol to maintain tight quality control over determination of VT_1_ and VT_2_ values was developed and included the following rules: (1) if, after concurrently viewing all graphs, an evaluator still thought that the VT value was indeterminate, then data for that subject were rejected. If the evaluators thought that data were usable, they then chose what they thought to be the most representative value. (2) The values determined by the three independent investigators were then compared by a fourth independent investigator. If the values determined by the evaluators were within 1 exercise stage (50 W or less than 15%), then values for the 3 investigators were averaged. (3) If values were within 15% of either of the initial investigators, then the VT values were averaged. Comparison values greater than 15% were removed from the analysis. (4) The appearance time of VT needed to be after the 75 W (after 4 min) warm-up of the exercise test or the data were rejected and were considered to be indeterminate. The data reported for the visual identification method include only those participants whose data met all the criteria. Evaluators rejected 57 for VT_1_ and 28 for VT_2_ based on our criteria. For the accurate comparison between the visual analysis and the automated analysis, we only used the paired matches.

### Statistical Analysis

Independent *t* tests were used when examining subject characteristics and sex comparison of exercise variables for VO_2_, W, HR, RER, and VE values at VT_1_ and VT_2_.

The mean differences of the subjective analysis and the automated analysis were examined using independent t test to test for differences between measures. The Bland–Altman analysis (Giavarina, 2015) assessed the limits of agreement between VT_1_ and VT_2_. Formal equivalence testing was conducted with the TOST approach with predefined equivalence bounds of ±25 (Schuirmann, 1987). The data were also analyzed by the intraclass correlation coefficients (ICC) to examine the relationships between the subjective analysis and the automated analysis.

For each subject, absolute VO_2_, relative VO_2_, W, HR, RER, and VE were determined at VT_1_ (average of V-slope for VT_1_ and ExCO_2_ methods), VT_2_ (average of V-slope for VT_2_ and ExVE methods), and peak, a 2-way factorial ANOVA was then used to examine main effects and interactions (VT × Group) for the sex difference study. If significance was found, the appropriate Holm-Sidak multiple comparison *post hoc* test was performed.

Data were analyzed and figures generated using MATLAB R2020a (The MathWorks, Inc., Natick, MA, United States) and GraphPad Prism (version 8.4.3, La Jolla, CA, United States) with statistical significance set at *p* < 0.05. Equivalence testing was conducted with the TOSTER package in the R statistical software (R: A Language and Environment for Statistical Computing, R Core Team, R Foundation for Statistical Computing, Vienna Austria, 2019).^1^ All data are reported as mean ± SD.

## Results

### Subject Characteristics and Sex Comparison of Exercise Variables at VT_1_ and VT_2_

Males and females were matched for age but different for mass and height (Table 1). Mean exercise variables for the overall subjects and sex difference are reported in Table 2. At peak, VT_1,_ and VT_2_, females had lower absolute O_2_ uptake, W, and ventilation and relative O_2_ uptake compared to men (*p* ≤ 0.04). HRs and RERs were not different at peak exercise for either group. However, HRs and RERs were lower in the male group compared to the female group at VT_1_ and VT_2_ (*p* ≤ 0.02).

**Table 2:** Mean values at peak, VT_1_, and VT_2_.

Study			VO_2_ (L/min)	VO_2_ (ml/kg/min)	Work rate (W)	HR (beat/min)	RER (VO_2_:VCO_2_)	VE (L/min)
Values at peak	Overall		3.3 ± 0.9	43.2 ± 7.7	303.3 ± 76.4	177 ± 10	1.28 ± 0.08	140.0 ± 37.0
	Male vs. Female	Males	3.8 ± 0.6	45.8 ± 7.1	339.9 ± 48.4	178 ± 8	1.29 ± 0.07	156.4 ± 31.1
		Females	2.4 ± 0.4	37.8 ± 5.8	225.9 ± 66.5	177 ± 13	1.28 ± 0.09	105.3 ± 21.2
		value of *P*	*<0.0001*	*<0.0001*	*<0.0001*	0.74	0.48	*<0.0001*
Values at VT_1_	Overall		1.9 ± 0.5	25.2 ± 4.5	156.2 ± 43.9	137 ± 15	0.99 ± 0.06	52.0 ± 11.3
	Male vs. Female	Males	2.1 ± 0.4	25.7 ± 4.8	172.3 ± 39.8	134 ± 14	1.00 ± 0.05	55.6 ± 10.4
		Females	1.6 ± 0.3	24.0 ± 3.4	120.2 ± 28.9	144 ± 15	0.97 ± 0.08	44.5 ± 9.4
		value of *P*	*<0.0001*	*0.04*	*<0.0001*	*<0.01*	*0.02*	*<0.0001*
Values at VT_2_	Overall		2.6 ± 0.6	33.3 ± 6.3	213.8 ± 53.2	155 ± 17	1.10 ± 0.06	77.0 ± 15.3
	Male vs. Female	Males	2.8 ± 0.5	34.3 ± 6.3	234.3 ± 45.3	151 ± 17	1.10 ± 0.06	82.2 ± 13.7
		Females	2.1 ± 0.4	31.3 ± 6.0	167.7 ± 39.3	162 ± 13	1.10 ± 0.08	65.8 ± 12.1
		value of *P*	*<0.0001*	*0.01*	*<0.0001*	*<0.01*	0.88	*<0.0001*

VO_2_, volume of oxygen; HR, heart rate; RER, respiratory exchange ratio; VE, ventilation; VT, ventilatory threshold.

### Relative Exercise Intensities at VT_1_ and VT_2_

**Figure 4:**
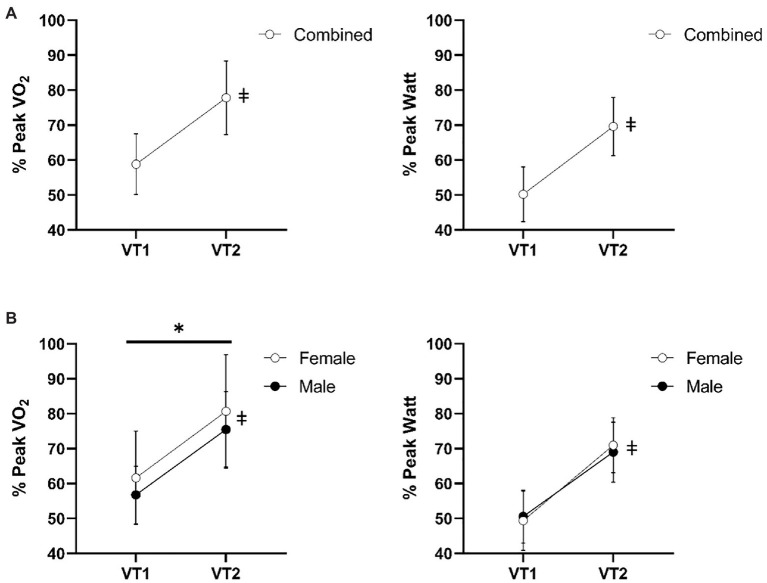
Exercise intensities at VT_1_ and VT_2_ relative to peak VO_2_ and peak W for the combined analysis **(A)** and sex differences **(B)**. ^*^Indicates group main effect differences *P* < 0.001. ǂ indicates significant main effect difference between VT_1_ and VT_2_ *p* < 0.0001. Data reported as means ± SD.

Exercise intensities at VT_1_ and VT_2_ relative to peak VO_2_ and peak W for the combined analysis in all subjects and sex differences are reported in Figure 4. In the combined analysis, VT_1_ occurred at 58% of peak VO_2_ and VT_2_ occurred at 79% of peak VO_2_ (*p* < 0.0001). Work rates at VT_1_ and VT_2_ were 50 and 69% of peak W (*p* < 0.0001). For the sex difference comparison, VT_1_ and VT_2_ occurred at a greater relative percentage of their peak VO_2_ for females (67 and 88%) compared to males (55 and 74%), main effect for group; *p* < 0.001. However, no differences were found for group expressed as a relative percentage of peak W. Both sexes had VT_1_ at 50% and VT_2_ at 70% of peak W (*p* ≥ 0.40).

### Comparison Between Visual and Automated

**Figure 5:**
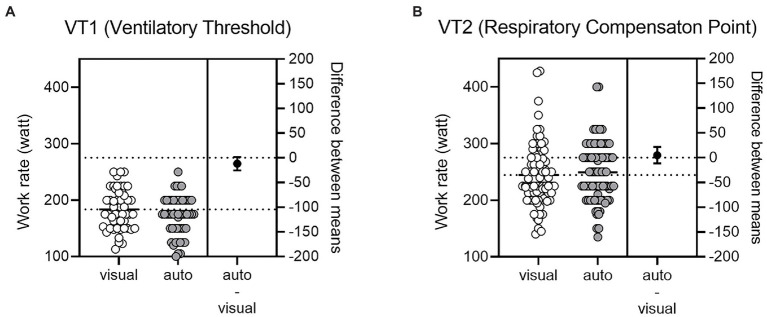
Bland–Altman analysis between visual and automated method. **(A)** VT_1_, **(B)** VT_2_.

Independent *t* tests for the comparison between the subjective analysis and the automated analysis found no difference for VT_1_ (*p* > 0.05) and VT_2_ (*p* > 0.05). Figure 5 illustrates the Bland–Altman plot. Bias for VT_1_ was 7.08 ± 12.2 W with the 95% Limits of Agreement from −16.99 to 31.06 W. Bias for VT_2_ was −2.2 ± 12.7 W with the 95% Limits of Agreement from −27.0 to 22.7 W. For VT_1_ and VT_2_ combined, the equivalence test was significant, *t*(132) = −9.389, *p* < 0.0001, given equivalence bounds of −25 and 25 (on a raw scale) and an alpha of 0.05. For VT_1_ and VT_2_ individually, the equivalence tests also were significant, *t*(51) = −4.513, *p* < 0.0001 and *t*(80) = 5.812, *p* < 0.0001, respectively, given equivalence bounds of −25 and 25 (on a raw scale) and an alpha of 0.05. Equivalence testing showed that the automated and visual measures were statistically equivalent (*p* < 0.0001) in all ways, each VT_1_ or VT_2_ and combined. Finally, a high degree of reliability was found between the subjective analysis and the automated analysis. The ICC between the subjective analysis and the automated analysis were 0.821 for VT_1_ and 0.830 for VT_2_ with a 95% confidence interval.

## Discussion

The advantages of a computerized method include faster, objective, and automated data analysis as well as improvements in reproducibility and repeatability. The aim of this study was to provide a novel, reliable, and computerized method for automatically identifying VT_1_ and VT_2_. We demonstrated that our method was able to determine ventilatory thresholds comparable to the visual analysis accomplished by 3 trained evaluators. We also determined the associated work rates expressed as absolute and relative submaximal VO_2_ and W and reported that sex differences exist for VT_1_ and VT_2_.

Others have compared various computerized methods for determining VT_1_ and demonstrated that regression-based methods provide considerably different results (Ekkekakis et al., 2008). For example, VT_1_ values detected by using 2 regression lines were significantly lower with weaker correlations compared to other computerized methods. Additionally, Pearson correlation coefficients in VO_2_ (L/min) between ExCO_2_ and V-slope for VT_1_ were 0.517 and 0.526 (i.e., a moderate positive relationship) in each sample group. In this study, Pearson correlation coefficient in VO_2_ (L/min) detected by ExCO_2_ and V-slope for VT_1_ using the parametric global optimization method was 0.81 (i.e., a strong positive linear relationship), which is a stronger correlation coefficient compared to using 2 regression lines (Ekkekakis et al., 2008). We also found that the strong positive relationship for Pearson correlation coefficient between computerized methods for determining VT_2_ (ExVE and V-slope for VT_2_) between ExVE and V-slope for VT_2_ means that the parametric global optimization method. In support of these, we found no difference between the subjective visual method by 3 trained evaluators and our automated method. Our novel and automated protocol may increase the methodological consistency in both research and clinical practice.

It has been reported that maximal aerobic capacity is associated with VT_1_ (59 to 65%) and VT_2_ (84 to 87%) and that maximal lactate steady state corresponds to VT_2_ (Pallarés et al., 2016; Cerezuela-Espejo et al., 2018). We found that when comparing to peak work rate, VT_1_ and VT_2_ were associated with 50 and 70% of peak W, respectively. These differences may be because of age and fitness of our study participants’ steady state vs. progressive exercise test. When we examined the ventilatory points in relation to peak VO_2_, the female group had greater relative VO_2_ associated with VT_1_ (67%) and VT_2_ (88%). Beaver et al. (1986) reported that VT_1_ occurred at 55% of peak VO_2_ and VT_2_ occurred at 75% of peak VO_2_ (Beaver et al., 1986) and a recent meta-analysis reported that VT_1_ occurs at 50 to 60% of peak VO_2_ (Galán-Rioja et al., 2020). These values are similar to ours as we report the whole sample was at 58 and 79% of peakVO_2_ for VT_1_ and VT_2_, respectively. As noted earlier, these differences in VT_2_ for the female group may be because of sensitivity of the carotid body ventilatory drive caused by body temperature, blood osmolarity, pH, K+, H^+^ buffering by bicarbonate, and the change in partial pressure of O_2_ (Galán-Rioja et al., 2020).

It has been reported that microgravity affects females and males differently (Mark et al., 2014). It is important to better understand these sex differences as the female representation in the astronaut corps is increasing, meaning more women will be eligible to fly in space than ever before. Under the microgravity environment, one of the sex-specific differences in exercise response is orthostatic intolerance caused by plasma volume loss and cardiovascular adjustments (Goel et al., 2014). Females generally have smaller body size, lower absolute, and relative aerobic fitness and are weaker in upper and lower body strength, which have implications for risk of fatigue and injury from muscle strains during EVA and emergency egress (Harm et al., 2001). Thus, understanding exercise countermeasures and the adaptations between sexes is of high relevance for the astronaut population. Our data are similar to others that report sex differences in gas exchange threshold for VT_1_. In this study, we also showed that VT_2_ differences occurred between males and females. This may be because of differences in breathing adjustments to chemosensitivity, thermoregulation, and menstrual cycle hormones (Beaver et al., 1986; Kilbride et al., 2003; Hayashi et al., 2012).

Others have suggested using exercise work rates above and below the ventilatory breakpoints for VT_1_ and VT_2_ for the prescription of exercise training to define exercise domains such as moderate, heavy-and severe exercise intensity domains (Beaver et al., 1986; Nattie and Li, 2012). This gives an individualized approach to prescribe exercise specific to the metabolic demands. Previous exercise training countermeasures during spaceflight or analogues have used a relative percentage of peak VO_2_ (e.g., continuous cycle exercise for 30 min at 75% of peak VO_2_ and interval treadmill sessions of 30 s to 4 min at nearly maximal intensity; Moore Jr et al., 2014; Ploutz-Snyder et al., 2018). However, the responses to these exercise prescriptions still have high variability for maintaining fitness. For example, Moore et al. reported that astronauts who have higher initial aerobic capacities are more prone to loss of cardiorespiratory fitness; however, the reason is unknown and may be because of the frequency, intensity, time, and progression of the exercise prescription (Moore et al., 2014). Our data suggest that sex should be considered when prescribing exercise countermeasures and the prescriptions could be further individualized by prescribing based on VT_1_, VT_2_, and peak VO_2_.

Notably, we acknowledge a limitation with our study. This includes not obtaining arterial lactate samples and blood gasses to confirm the metabolic and ventilation breakpoints. Further validation should include these measurements.

## Conclusion

In summary, the new automated method has been shown to identify inflection points in each of the variables used to reliably determine VT_1_ and VT_2_. Furthermore, we show that sex influences the VT_1_ and VT_2_ in members of the US astronaut corps. Detection of both VT_1_ and VT_2_ and their associated absolute and relative work rates may provide valuable information regarding crewmembers’ ability to exercise on the ISS and to perform EVAs and lunar exploration. Lastly, accurately tracking fitness pre-, in-, and post-flight is of importance for guidance on the efficacy of exercise training prescriptions as countermeasures.

## Data Availability Statement

The datasets presented in this article are not readily available because the study dataset are not publicly available due to privacy laws and other restrictions. Requests to access the datasets should be directed to KK, kyoungjae.kim@nasa.gov.

## Ethics Statement

The studies involving human participants were reviewed and approved by NASA Johnson Space Center. The patients/participants provided their written informed consent to participate in this study.

## Author Contributions

KK: conceptualization, methodology, software, data curation, writing – original draft, writing – review and editing, and visualization. ER: methodology, validation, formal analysis, data curation, writing – original draft, and writing – review and editing. BP and DF: validation and writing – review and editing. MY: validation, formal analysis, writing – original draft, and writing – review and editing. MD: investigation, resources, writing – review and editing, supervision, project administration, and funding acquisition. All authors contributed to the article and approved the submitted version.

## Funding

This work was supported by the NASA Habitations System Account.

## Conflict of Interest

The authors declare that the research was conducted in the absence of any commercial or financial relationships that could be construed as a potential conflict of interest.

## Publisher’s Note

All claims expressed in this article are solely those of the authors and do not necessarily represent those of their affiliated organizations, or those of the publisher, the editors and the reviewers. Any product that may be evaluated in this article, or claim that may be made by its manufacturer, is not guaranteed or endorsed by the publisher.

## Footnotes

^1^ https://www.R-project.org
